# Assessing the Left Ventricular Systolic Function at the Bedside: The Role of Transpulmonary Thermodilution-Derived Indices

**DOI:** 10.1155/2011/927421

**Published:** 2011-07-27

**Authors:** Gerardo Aguilar, F. Javier Belda, Carlos Ferrando, José Luis Jover

**Affiliations:** ^1^Departamento de Anestesiología y Cuidados Críticos, Hospital Clínico Universitario de Valenica, 46010 Valencia, Spain; ^2^Departamento de Anestesiología, Hospital Virgen de los Lirios, Alcoy, 03804 Alicante, Spain

## Abstract

Evaluating the systolic function of the left ventricle (LV) is important in the hemodynamic management of the critically ill patients with circulatory failure. Echocardiography is considered the standard monitor for estimating the LV function at the bedside in the intensive care unit. However, it requires a trained operator and is not a real-time monitoring tool. For monitoring of the systolic function, the pulmonary artery catheter has been the gold standard for a long time. However, now there are alternatives to this device, with transpulmonary thermodilution being one of them. This paper provides an overview of the usefulness of the transpulmonary thermodilution-derived indices for assessing systolic function at the bedside.

## 1. Introduction

Cardiovascular monitoring is essential for diagnostic and therapeutic management of critically ill patients and assessing the systolic function of the left ventricle (LV) is a key component in this strategy. 

Echocardiography has become the standard tool for measuring LV ejection fraction (LEVF) at the bedside in the intensive care unit (ICU). This type of monitoring gives the physician a rapid and accurate etiologic diagnosis of the cause of hemodynamic instability in the critically ill patient. Thus, a hemodynamically unstable patient is a good reason to perform a cardiac ultrasound [1]. However, the use of echocardiography requires an expensive device and a trained operator. Additionally, conventional echocardiography cannot be considered as continuous monitoring, and although there is a commercially available continuous model, it has not received widespread acceptance.

The pulmonary artery catheter (PAC) has been the gold standard for monitoring of systolic function for decades, but concerns have been raised about its safety and the clinical usefulness of the data it provides [2–4]; thus, alternative monitoring methods have been evaluated.

More recently, the transpulmonary thermodilution technique with single thermal indicator (incorporated into the PiCCO monitor, Pulsion Medical System, Munich, Germany) was proposed as a “less invasive” hemodynamic monitoring system for critically care patients. The system provides intermittent (transpulmonary thermodilution-derived) and continuous (pulse contour-derived) assessment of cardiac output and estimations of intrathoracic volumes (intrathoracic blood volume, global-end diastolic volume, and extravascular lung water). Accuracy of cardiac output calculation using the PiCCO system has been demonstrated in several clinical studies [5–9] and intrathoracic blood volume (blood volume contained in the heart and in the intrathoracic vessels) and global end-diastolic volume (the volume of blood contained in the four cardiac chambers at the end of the diastole) have been shown to provide reliable and more sensitive estimates of cardiac preload than cardiac filling pressures obtained with the central venous and pulmonary artery catheters [10–17]. On the other hand, the use of the transpulmonary thermodilution arterial catheters do not increase the risk of complications when compared with the commonly used short peripheral arterial catheters or pulmonary artery catheters [18].

The PiCCO system also provides two transpulmonary thermodilution-derived indices of cardiac systolic function: the cardiac function index (CFI) and the global ejection fraction (GEF) which are automatically calculated by the monitor.

### 1.1. Calculation of CFI and GEF by Transpulmonary Thermodilution

A thermistor placed into a femoral, brachial, or axillary arterial catheter measures the downstream temperature changes induced by the injection of a cold saline bolus into the superior vena cava. The monitor calculates cardiac output (CO) from the thermodilution curve, using the Stewart-Hamilton algorithm, and also the mean transit time (MTt) and the exponential down slope time (DSt). The result of the product of CO times MTt is the intrathoracic thermal volume (ITTV):

(1) ITTV=CO×MTt.

And the product of CO times DSt is the pulmonary thermal volume (PTV):

(2) PTV=CO×DSt.

The difference between ITTV and PTV is the global end-diastolic volume (GEDV) which represents the volume of blood contained in the four chambers:

(3) GEDV=ITTV−PTV=CO×(MTt−DSt) (mL).

The CFI is defined as the ratio of cardiac output to the global end-diastolic volume:

(4) $$\text{CFI}=\frac{\text{CO}}{\text{GEDV}}\;\left({\min\;}^{-1}\right).$$

The GEF is defined as the ratio of the stroke volume (SV) to the quarter of the global end-diastolic volume:

(5) $$\text{GEF}=\frac{\text{SV}}{\left(\text{GEDV}/4\right)}\;\left(\text{\%}\right).$$

CFI and GEF are, therefore, global ejection phase indices since they are the ratio of CO or stroke volume to the global end-diastolic volume of the heart. Therefore the difference between the two indices is that GEF takes into account the heart rate and stoke volume, and the GEF only considers the stroke volume. These indices are obtained very easily by the physician at the bedside while only an experienced operator can get similar information using echocardiography [17]. 

 The purpose of this paper is to review the different studies that assess the accuracy of these transpulmonary thermodilution-derived indices for the estimation of left ventricular systolic function in the ICU patients at the bedside.

## 2. Evaluation of the Transpulmonary Thermodilution-Derived Indices with the Echocardiography

In 2004, Combes et al. used transesophageal echocardiography to compare these indices with left ventricular fractional area of change (LVFAC) [17]. They studied 33 adult ICU patients with no isolated right ventricular dysfunction in a prospective, open clinical study. During the measurements, echocardiography identified 3 patients with isolated right ventricular failure, in which transpulmonary thermodilution underestimated LVFAC. In the results, significant correlations were established between LVFAC and CFI (*r* = 0.87, *n* = 30, *P* < 0.0001) or GEF (*r* = 0.82, *n* = 30, *P* < 0.0001). The mean differences between LVFAC and LVFAC estimated with CFI or GEF were 0.8 ± 8.5% (range −17 to 14%) and 0.8 ± 9.0% (range −21 to 19%), respectively. Area under the receiver operating characteristics curves for the estimation of LVFAC ≥ 40% using CFI or GEF was 0.92. Values of CFI > 4 and GEF > 18% estimated LVFAC ≥ 40% with respective sensitivities of 86 and 88% and specificities of 88 and 79%. Additionally, significant correlations were found between changes of LVFAC and CFI and GEF over time. The authors concluded that in mechanically ventilated ICU patients, GEF and CFI provide reliable estimations of LV systolic function, but may underestimate it in cases of isolated right ventricular failure.

Five years later, Jabot et al. conducted a prospective study in 48 medical ICU patients with acute circulatory failure, to assess whether CFI could actually behave as an indicator of left ventricular systolic function [19]. For this purpose they tested if CFI fulfilled the following criteria: (1) it increased with inotropic stimulation (dobutamine infusion, *n* = 24); (2) it was not altered by fluid loading (500 mL of saline, *n* = 24); (3) it correlated with the echocardiographic left ventricular ejection fraction (LVEF). The authors simultaneously measured LVEF (monoplane or biplane Simpson method) and CFI at baseline, and after saline and dobutamine administration. Volume expansion altered neither LVEF (47 ± 11% to 47 ± 11%) nor CFI (4.5 ± 2.2 to 4.5 ± 2.1 min^−1^), dobutamine infusion significantly increased LVEF (percentage of change: 32 ± 28%) and CFI (percentage of change: 29 ± 22%). Considering the effects of dobutamine, there was a significant correlation between the changes in CFI and the changes in LVEF (*r* = 0.65, *P* = 0.0001). Finally, a CFI < 3.2 min^−1^ predicted an LVEF of ≤35% with a sensitivity of 81% and specificity of 88%. The authors concluded that CFI fulfilled the criteria required from a clinical indicator of left ventricular global systolic function and accurately tracked the effects of inotropic therapy.

**Figure 1:**
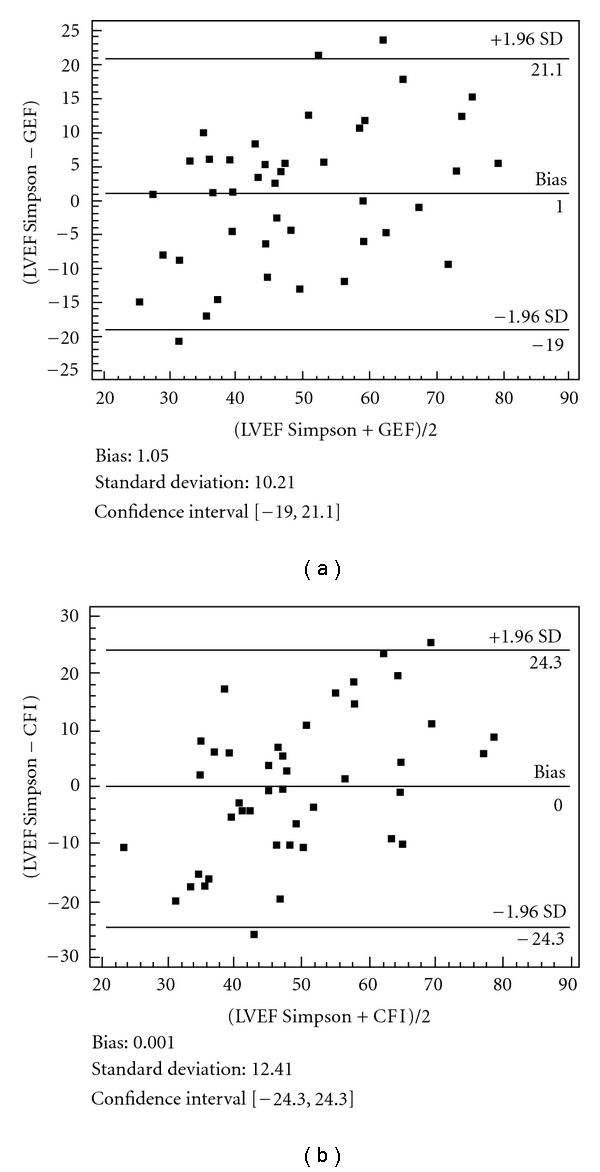
Bland-Altman analyses of agreement between GEF (a) or CFI (b) and the LVEF measured by the Simpson method. The central line is the mean difference (bias) between the two methods whereas the outer lines represent the two SD limits of agreement. From Belda et al. [18] with permission.

Finally, our group conducted a prospective clinical study with 35 ICU patients, excluding those with severe changes in contractility and in nonsinus rhythm [20]. We compared these indices with the left ventricular ejection fraction obtained by transthoracic echocardiography. In the results we found significant correlations between the left ventricular ejection fraction and the GEF (*r* = 0.79, *P* < 0.001) and the CFI (*r* = 0.66, *P* < 0.001). The mean differences between LVEF and LVEF estimated with GEF or CFI (Figure 1) were 1.05 ± 10.2% (range −19 to 29.1%) and 0.001 ± 12.4% (range −24.3 to 24.3%), respectively. For predicting an LVEF of less than 40%, the area under the curve was 0.879 for the GEF and 0.805 for the CFI. Furthermore, a GEF of less than 13.5% and a CFI of less than 3.15 min^−1^ predicted an LVEF of less than 40% with sensitivities of 97% and 96% and specificities of 85% and 77%, respectively. We concluded that in patients without marked changes in contractility, the GEF and the CFI offer a reliable and simple way to assess the left ventricular ejection fraction.

## 3. Identifying Cardiac Dysfunction in Acute Heart Failure and Septic Patients

In 2009, Ritter et al. designed an observational study comparing the cardiac function of patients with acute heart failure (AHF) or sepsis using the pulmonary artery catheter and the PiCCO technology [21]. Twelve patients with AHF and nine patients with severe sepsis or septic shock had four simultaneous hemodynamic measurements by PAC or PiCCO during a 24-hour time period. In the results, compared to septic patients, AHF patients had significantly lower cardiac index (CI), CFI, GEF, mixed venous oxygen saturation (SmvO_2_), and pulmonary vascular permeability index (PVPI), but higher pulmonary artery occlusion pressure (PAOP). The mean values in the groups for sepsis and AHF were, respectively: CI (PiCCO) 4.2 versus 2.9 L·min^−1^·m^−2^, CI (PAC) 4.3 versus 2.7 L· min^−1^·m^−2^, CFI 6.2 versus 2.7 min^−1^, GEF 23 versus 13%, SmvO_2_ 69 versus 54%, PVPI 2.8 versus 2.6 and PAOP 17 versus 20 mmHg. There were no significant differences between the two groups in the extra lung water index (ELWI, mean values): 16.7 versus 15.5 mL·kg^−1^. Additionally, PAOP did not correlate with ELWI and PVPI either in septic shock or in AHF patients. 

All patients with a CFI less than 4.5 min^−1^ had a SmvO_2_ not greater than 70%. In both groups, the CFI show a weak but statistically significant correlation with the left ventricular stroke work index (sepsis: *r* ^2^ = 0.30, *P* < 0.05; AHF: *r* ^2^ = 0.23, *P* < 0.05) and the cardiac power (sepsis: *r* ^2^ = 0.39, *P* < 0.05; AHF: *r* ^2^ = 0.45, *P* < 0.05). The authors concluded that in critically ill medical patients, assessment of cardiac function using the transpulmonary thermodilution technique is an alternative to the PAC. Furthermore, a low CFI identifies cardiac dysfunction in both AHF and septic patients.

## 4. Conclusions

The transpulmonary thermodilution-derived indices, cardiac function index (CFI) and global ejection fraction (GEF), can be considered as useful indicators of left ventricular global systolic function. In fact, both could help the physician identify, easily and at the bedside, alterations in the left ventricular ejection fraction. On the other hand, normal values of these indices indicate a good systolic function and could avoid the need for immediate echocardiographic evaluation.

## Abbreviations

AHF: Acute heart failure
CFI: Cardiac function index
CI: Cardiac index
CO: Cardiac output
DSt: Downslope transit time
ELWI: Extra lung water index
GEDV: Global end-diastolic volume
GEF: Global ejection fraction
ICU: Intensive care unit
ITTV: Intrathoracic thermal volume
LV: Left ventricle
LVEF: Left ventricular ejection fraction
LVFAC: Left ventricular fractional area of change
MTt: Mean transit time
PAC: Pulmonary artery catheter
PAOP: Pulmonary artery occlusion pressure
PiCCO: Pulse-induced contour cardiac output
PTV: Pulmonary thermal volume
PVPI: Pulmonary vascular permeability index
SV: Stroke volume
SmvO_2_: Mixed venous oxygen saturation.
